# Cardamonin protects against iron overload induced arthritis by attenuating ROS production and NLRP3 inflammasome activation via the SIRT1/p38MAPK signaling pathway

**DOI:** 10.1038/s41598-023-40930-y

**Published:** 2023-08-23

**Authors:** Shaocong Li, Qi He, Baihao Chen, Jiaxu Zeng, Xiangyun Dou, Zhaofeng Pan, Jiacong Xiao, Miao Li, Fanchen Wang, Chuyi Chen, Yuewei Lin, Xintian Wang, Haibin Wang, Jianfa Chen

**Affiliations:** 1 First School of Clinical Medicine, Guangzhou University of Chinese Medicine, 12 Jichang Road, Baiyun Area, , Guangzhou, 510405 People’s Republic of China; 2 The Laboratory of Orthopaedics and Traumatology of Lingnan Medical Research Center, Guangzhou University of Chinese Medicine, Guangzhou, 510405 People’s Republic of China; 3 Department of Orthopaedics, The First Affiliated Hospital, Guangzhou University of Chinese Medicine, 16 Jichang Road, Baiyun Area, Guangzhou, 510405 People’s Republic of China

**Keywords:** Cell biology, Cell signalling

## Abstract

Iron homeostasis plays an essential role in joint health, while iron overload can cause damage and death of cartilage cells. Cardamonin (CAR) is a substance found in the fruit of the chasteberry plant and has anti-inflammatory and anti-tumor activities. We first administered iron dextran (500 mg/kg) intraperitoneally to establish an iron overload mouse model and surgically induced osteoarthritis. The extent of OA and iron deposition were assessed using Micro-ct, Safranin-O/fast green staining, H&E staining, and Prussian Blue 10 weeks later. We administered primary chondrocytes with Ferric Ammonium Citrate (FAC) to evaluate the chondrocyte changes. Chondrocytes were identified in vitro by toluidine blue staining, and chondrocyte viability was evaluated by CCK-8. The rate of apoptosis was determined by Annexin V-FITC/PI assay. The mechanism of action of CAR was verified by adding the SIRT1 inhibitor EX527, and the expression of SIRT1 and MAPK signaling pathways was detected by Western blot. Iron overload also promoted chondrocyte apoptosis, a process that was reversed by CAR. In addition, CAR reduced NLRP3 inflammasome production via the SIRT1-MAPK pathway, and the SIRT1 inhibitor EX527 inhibited the treatment of OA by CAR.CAR inhibited cartilage degeneration induced by iron overload both in vivo and in vitro. Besides, our study showed that iron overload not only inhibited type II collagen expression but also induced MMP expression by catalyzing the generation of NLRP3 inflammasome. Our results suggest that CAR can treat KOA by promoting SIRT1 expression and inhibiting p38MAPK pathway expression to reduce the production of NLRP3 inflammasome vesicles.

## Introduction

Knee osteoarthritis (KOA) is the most common degenerative joint disease in middle-aged and elderly individuals, especially women in menopausal period. It affects over one billion people in nearly two hundred countries worldwide, and the number of sufferers continues to rise, with over 60 million people in the United States expected to suffer from the disease by 2030^1^. KOA can restrict areas including the subchondral bone, synovium, and articular cartilage, and its main symptoms include stiffness, pain, and swelling of the knee and hip joints, thus limiting a patient's limb movement and reducing the quality of life. The main treatment modalities for KOA are oral non-steroidal anti-inflammatory drugs (NSAIDs)or artificial joint replacements, the former of which is sometimes not as effective as it should be, and the latter of which is more economically burdensome. Therefore, the search for new ideas for arthritis treatment is now controversial.

Previously, KOA was generally thought to be a disease caused by long-term mechanical damage to joints. However, with advancements in related research, the causes of arthritis have been identified to be possibly related to blood type, inflammation, age, and metabolic factors . In addition, whole-body iron overload and cartilage iron deposition are key factors in KOA^2^. Studies have reported that excessive cellular iron will affect the expression of iron-transport-related proteins and promotes the production of excessive lipid peroxides through the Fenton reactionl. This eventually leads to membrane lipid peroxidation, mitochondrial dysfunction, cellular nucleic acid and protein damage, and cell death^3^. Oxidative stress and mitochondrial dysfunction have been demonstrated as important factors in the development of KOA^4^.Thus, improvement of iron overload is widely believed to have a significant therapeutic effect on arthritis caused by iron overload and iron deposition in the joints^5^.

For the past 40 years, the iron chelator, deferoxamine (DFO), has been the most widely used treatment for iron overload.However, DFO often causes side effects during clinical treatment, including hypotension and palpitations, and its long-term use can significantly reduce the quality of life of patients^6^. Chinese herbal medicines (CHMs) have demonstrated great potential in treating various diseases, including KOA and iron overload^7^. Therefore, exploring CHMs for the treatment of KOA is a viable approach. *Cardamonin (CAR),* a substance present in the fruits of Ligustrum species, has anti-inflammatory and antitumor activities.It is also effective in inhibiting arthritis and reducing plasma levels of inflammatory factor^8–11^. In this study, we aimed to investigate the effect of CAR on iron overload-induced arthritis in vitro and in vivo and to explore its mechanism for treating KOA.

Sirtuins is the family of proteins involved in various cellular functions^12^. SIRT1, a member of the sirtuin family, is a nicotinamide adenine dinucleotide-dependent histone deacetylase with anti-inflammatory, antioxidant, and anti-apoptotic effects^13^. Some studies have reported that SIRT1 inhibits proinflammatory responses in articular chondrocytes^14^. SIRT1 is also involved in the regulation of cardiomyocyte and hepatocyte damage caused by ROS accumulation induced by iron overload. Furthermore, it protects fibroblasts from oxidative stress-induced apoptosis in patients with psoriasis and restores mitochondrial function and redox balance by down-regulating the p38MAPK/NLRP3 signaling pathways^15^. NLRP3 is a complex of multiple proteins that can activate inflammatory responses^16^. It is considered to be one of the main triggers of KOA^17^. SIRT1 activation inhibits NLRP3 inflammasome activation and subsequent IL-1βsecretion^18^, whereas SIRT1 knockdown enhances NLRP3 inflammasome activation in cultured endothelial cells^19,20^. Therefore, we investigated whether CAR alleviates the oxidative stress damage caused by iron overload through SIRT1.

## Materials and methods

### Materials and reagents

Penicillin/streptomycin (P/S, 15140122), trypsin and TRIzol Reagent were provided by Thermo Fisher Scientific. Servicebio Technology offered DMEM/F12(DMEM/F12, 11320033)medium, PBS, 1% toluidine blue staining solution, foetal bovine serum (FBS, 10100147) (Scoresby, Australia). Sigma-Aldrich supplied Ferric Ammonium Citrate (FAC,F5879), deferoxamine (DFO, D9533),Iron Dextran, and Collagenase. Deset Biotech supplied APT. GlpBio Technology offered Cell Counting Kit (CCK8, BS350A)and paraformaldehyde (BL539A). Beyotime Biotechnology offers RIPA buffer(RIPA, P0013B), BCA detection kit (BCA, P0012S) , DS-PAGE gel preparation kit(P0690). The ECL Chemiluminescent Substrate Kit was from Biosharp Biotechnology. Primary antibody was from Santa Cruz.And EX527(EX527,HY-15452) was purchased from MCE (MedChemExpress, America). Cardamonin was purchased from Chengdu Refinebio Biological Technology(China). DAB staining kit was purchased from Aladdin(China).

### Cell isolation and culture

We removed knee cartilage from 3 weeks old male C57/BL6 mice, then minced it to approximately 1 mm3, and washed it three times with PBS,then digested it with 0.25% trypsin and 0.25% collagenase solution.After collecting the cells, they were placed in a 5% CO_2_ cell incubator and cultured in DMEM/F12 medium containing 10% fetal bovine serum and 1% P/S(Penicillin–Streptomycin).

### Cell treatment

To test the effect of CAR on iron overload-induced arthritis, we intervened chondrocytes with 100 μm FAC. And we intervened MOD, PC, CAR-L, CAR-H with DFO100 μm, 2.5 μm and 5 μm CAR, respectively. In addition, to investigate whether CAR inhibits NLRP3 inflammasome expression and chondrocyte apoptosis by regulating the SIRT1/p38MAPK pathway, we divided the cells into NC, MOD + EX527, CAR, and CAR + EX527 groups, and treated the EX527 group with 10 μm SIRT1 inhibitor EX-527 and 100 μm fac. 100 μm fac with 5 μm CAR treated CAR group. 10 μm EX-527 and 100 μm fac and 5 μm CAR treated CAR + EX527 group.

### CCK8 cell viability assay

The cell counting Kit-8 (CCK8, GlpBio, USA) was adopted to evaluate the viability of chondrocytes.96*well plates were seeded with chondrocytes at a density of 4 × 103 per well, the cells were then cultured in DMEM/F12 medium (Servicebio, China) with 10% foetal bovine serum (FBS, Gibco, USA), 1% penicillin–streptomycin (Gibco, USA). The viability of chondrocytes was examined by intervening with different doses of CAR(0, 2.5, 5, 10, 20, 30 and 40 μM respectively) of CAR (DeSiTe Biological Technology, China) for 48 h at the atmosphere of 37 °C. Each well was then incubated overnight at 37 °C, lucifugally, with 10 μl CCK8 solution.Finally, microplate reader (Thermo Scientific, USA) was used to read the absorbance at 450 nm. Different groups' optical density values represented their viability.Upon detection of the appropriate concentration of CAR, the cells were coteated with 100 μM FAC to simulate an iron overload environment. Cell culture method was the same as mentioned above with the CAR (0, 2.5,5, 10, 20, 30,and 40 μM, respectively) treatment. The optimal therapeutic CAR concentration is obtained by repeated times.

### Toluidine blue staining

We washed the cells three times with PBS, then 1% toluidine blue staining solution (Servicebio, China) was added for 5 min at room temperature, and the cells were washed with PBS for 15 min , then aspirated and observed under the microscope (Leica, Germany).

### Assessment of intracellular ROS

To evaluate the levels of ROS in chondrocytes,cell ROS concentrations were assessed by the manufacturer's guidelines using the ROS assay kit. We seeded primary cells at a concentration of 1 × 105 on 24-well plates . After 24 h,we treated the cells for 48 h using the method described above. After washing the chondrocytes 3 times with serum-free medium,we incubated chondrocytes in the dark for 20 min with 10 μM dichloro‑dihydro‑ fluorescein diacetate (DCFH‑DA) or 5 μM C11 BODIPY .After washing the cells with serum-free medium, a fluorescent microscope made by Leica (Wetzlar, Germany) was used for examination. The excitation wavelength of the microscope was 488 nm, the emission wavelength was 525 nm, and the magnification was 100×. Cells were also evaluated via FACS LSRFortessaTM flow cytometer (BD Biosciences, Franklin Lakes, NJ) after the same treatment.

### Evaluation of apoptosis

We used the Annexin V-FITC/PI kit to detect apoptosis.Before observed under the FACS-Canto™ II flow cytometer (BD Biosciences, USA).Cells were treated with CAR and washed 3 times with PBS afterwards, and treated with Annexin V-FITC and PI for 20 min at 37 °C away from light.

### Western blot analysis

Cells were first washed three times with PBS, then mixed with 2% protease inhibitor (Beyotime, China) using RIPA buffer, and protein concentrations were measured with a BCA assay kit (Beyotime, China).Then, 8% or 10% sodium dodecyl sulfate–polyacrylamide gels (Produced by DS-PAGE Gel Preparation Kit, Beyotime Biotechnology, China) were prepared for electrophoresing. 10 µg of each sample was transferred to polyvinylidene difluoride membranes after electrophoresis(Millipore, USA).After that, the membranes were to be washed three times, and incubated with antibodies against MMP3 (1:1000, Affinity Biosciences, USA), Bax (1:1000, Affinity Biosciences, USA), BCL2 (1:1000, Affinity Biosciences, USA), Col II (1:1000, Wanleibio, China), CASP1(1:1000, Wanleibio, China),IL-1β-Mature(1:1000, Wanleibio, China), IL-18(1:1000, Wanleibio, China) , NLRP3(1:1000, Affinity Biosciences, USA), SIRT1(1:1000, Wanleibio, China), p-p38(1:1000, Wanleibio, China), p38(1:1000, Affinity Biosciences, USA), and β-actin (1:2000, Affinity Biosciences) at 4 °C overnight. Soon afterwards, peroxidase-conjugated was used to treat the membranes secondary anti-rabbit IgG antibody (1:1000, Affinity Biosciences, USA) for about 1 h at room temperature.The ECL chemiluminescent substrate kit was used to visualized the signals chemiluminescently. The protein bands’ semi-quantitative depends on the gel imaging system (Bio-Rad, USA). β-actin served as a loading control.

### Animal experiment

Sixty 6-week-old male wild-type C57BL/6J mice (purchased from the Institute of Model Animals, Nanjing University, SCXK (YUE) 2018-0034) were selected. All experimental animals were housed in the experimental animal center of Guangzhou University of Traditional Chinese Medicine at SPF level with constant temperature (22–25 °C) and 55–60% humidity, and were fed and watered freely. Sixty mice were randomly divided into 5 groups (SHAM MOD PC CAR-L CAR-H) of 10 animals each, and the animals were fed for 10 weeks. At the age of 8 weeks, the mice in the sham group were intraperitoneally injected with normal saline once a week, and the other groups’ mice were injected intraperitoneally with iron dextran (iron dextran injection 500 mg/kg) once a week. At 10 weeks of age, except the sham operation group, the mice in the other groups were operated on the right leg of the DMM model (the mice were anesthetized with pentobarbital sodium 30 mg/kg). After surgery, the mice were anesthetized and awakened, and fed and watered normally. The PC group was given NAC (100 mg/kg) by gavage for 8 weeks after surgery, and the CAR-L and CAR-H groups were given 3 mg/kg and 5 mg/kg CAR^21^ for 8 weeks, respectively. At 18 weeks of age, the injection of iron dextran was discontinued, over-anesthetized and executed (mice were executed with 300 mg/kg sodium pentobarbital) , and the knee joint of the operated leg was separated and the surrounding soft tissues were removed as cleanly as possible and placed in a 4% paraformaldehyde solution for 24 h of fixation. These experiments were approved by the Review Board of The First Affiliated Hospital of Guangzhou University of Chinese Medicine (no. TCMF1–2,021,029; May 20, 2021) , all experiment were performed in accordance with the relevant guidelines and regulations of the Review Board of The First Affiliated Hospital of Guangzhou University of Chinese Medicine.

### Micro-computed tomography (MICRO-CT) analysis

We fixed the knee samples in 4% paraformaldehyde, scanned them using Skyscan 1172 (Bruker, Belgium), and evaluated the data using a customized analysis program (CTAn, Skyscan).The 3D reconstruction and the data analysis were completed using direct 3D measuring techniques, the BV/TV, Tb. Sp, Tb. Th,Tb. N parameters were calculated.

### Immunohistochemistry

Medial joint compartments of previously decalcified samples were sectioned and decalcified on 5 µm sagittal sections. Antigens were recovered and sections were incubated with primary antibody against NLRP3 followed by biotinylated goat anti-rabbit secondary antibody. Sections were incubated with DAB for 10 min. Immunohistochemical staining images were analyzed using Image J software (Wayne Rasband, National Institutes of Health, USA).

### Histology assay

All specimens were fixed by 4% polymethanol and then placed in 14% EDTA for 10 days for decalcification. After decalcification, specimens were wrapped in paraffin and cut into 5-μm slices.The slices were stained with Safranin O/fast Green, Prussian Blue, hematoxylin–eosin (HE) and scanned by a Panoramic Midi digital slide scanner (3DHISTECH Ltd., Hungary).

### Statistical analysis

All measured values were expressed as mean ± standard deviation (x ± s) using SPSS 19. 0 statistical software, and the data were tested by independent sample t-test, and P < 0. 05 indicated that the differences were statistically significant.

### Ethics approval and consent to participate

All animal experiments were approved by the Review Board of The First Affiliated Hospital of Guangzhou University of Chinese Medicine (Ethic NO. TCMF1-2021029). The study is reported in accordance with ARRIVE guidelines (https://arriveguidelines.org).

## Result

### CAR attenuates iron overload-induced KOA injury in mice

**Figure 1:**
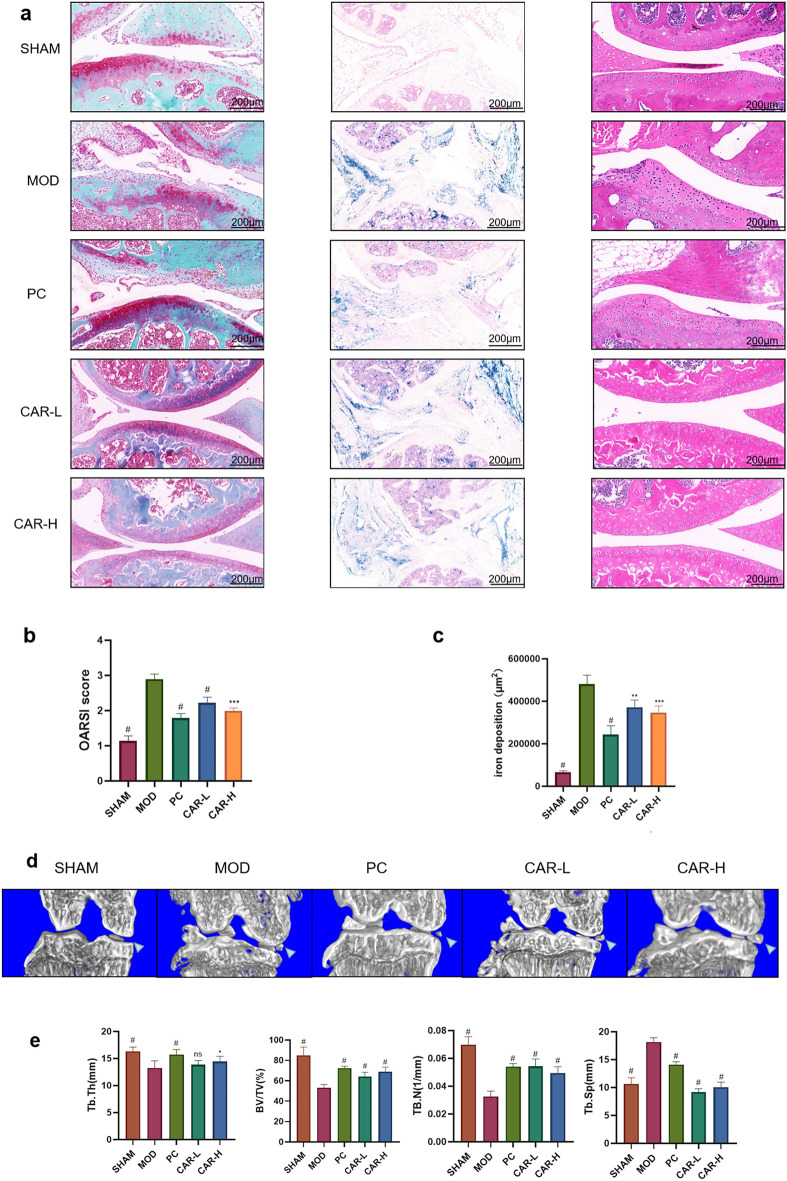
CAR reduces the severity of KOA in the knee joint. (**a**) Representative graphs of Safranin-O/Fast Green staining (left), Prussian blue staining (middle), H&E staining (right) (Scale bar = 200 μm). (**b**) OARSI scores of Safranin O/ Prussian blue staining. (**c**) Iron deposition in the knee joint of mice. (**d**) Representative 3D images of the knee joint. Quantitative analysis of (**e**) BV/TV, Tb.Th, Tb. N, Tb.Sp. (Each group was compared with the MOD group. data are presented as standard deviation ± mean; # *P* < 0.0001, *** *P* < 0.001, ** *P* < 0.01, * *P* < 0.05 vs. Mod).

**Figure 2:**
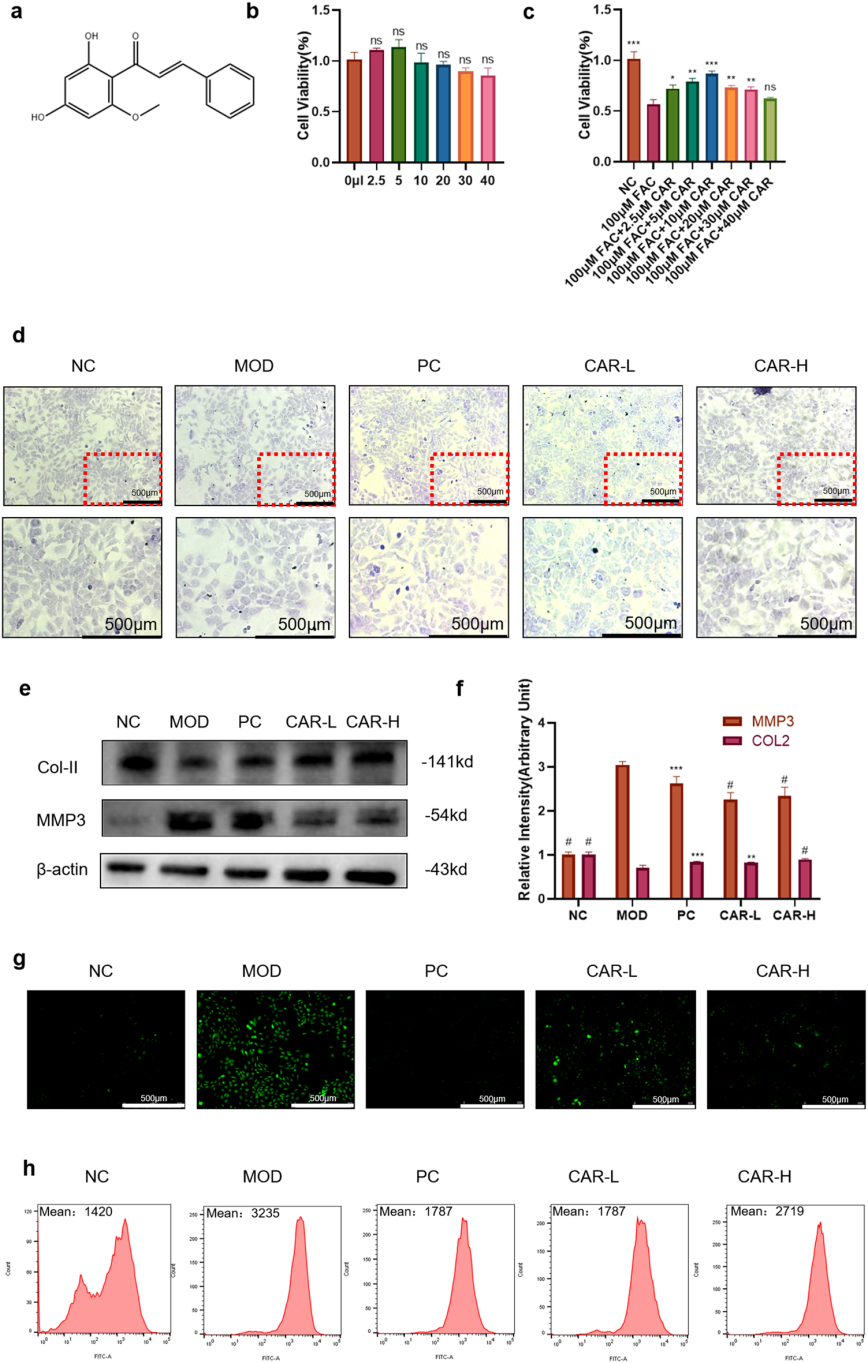
Effect of CAR on chondrocyte viability and ability to produce type II collagen in normal or iron-overloaded chondrocytes. (**a**) Chemical structure of Cardamonin. (**b**,**c**) Changes in chondrocyte viability after CAR intervention at concentrations of 0, 2.5, 5, 10, 20, 30, and 40 μm, with or without 100 μm FAC. (**e**,**f**) Chondrocytes were treated with 100 μm FAC and co-cultured with 0, 2.5, and 5 µM CAR to verify COL2, and MMP3 protein levels using protein blotting. Statistical results are shown in the bar graph. The results indicate that CAR reduced FAC-induced chondrocyte damage in a concentration-dependent manner. (**d**) The ability to synthesize type II collagen was measured using toluidine blue, the darker the color, the greater the ability. (**g**) The fluorescence intensity of ROS was analyzed by flow cytometry. (**h**) The fluorescence microscope images of ROS (data are presented as standard deviation ± mean; # *P* < 0.0001, *** *P* < 0.001, ** *P* < 0.01, * *P* < 0.05 vs. Mod).

It is a vital issue taht whether CAR(Fig. 2A) could alleviate KOA progression. We divided mice into five groups: SHAM, FAC, NAC, CAR-L, and CAR-H. All mice were subjected to DMM surgery, and mice outside the SHAM group were given intraperitoneal injection of iron dextran for modeling, while the NAC group, CAR-L, and CAR-H were gavaged with corresponding drugs. Prussian blue staining indicated that a large amount of iron ions were obviously present in the joint cavity after the injection of iron dextrose (Fig. 1A,C); As we can see, significant iron deposition could be spotted in cartilage and subchondral bone, staining with safranin-O/fast green showed that mice injected with iron dextrose had thinner articular cartilage thickness, more severe wear, and upward migration of subchondral bone compared with others, and the CAR gavage group could improve the above situation (Fig. 1A,B).Further more, H&E staining has showed that chondrocytes of articular cartilage in the model group were disordered. And the OARSI score could also be consistent with the above findings. To visualize the subchondral bone morphology in mice more, we used Micro-ct to model the mouse knee joint in three dimensions (Fig. 1D). Cross-sectional images showed that after FAC intervention, all groups of mice showed significant osteophytes in the tibia; compared with the SHAM group, the DMM group showed relatively thin subchondral bone plates, slender and disorganized bone trabeculae, and compressed subchondral bone (Fig. 1D). Treatment with NAC and CAR reduced the structural changes of bone trabeculae and decreased the degree of osteophytes. We also used 3D μCT with subchondral bone morphology analysis to examine the protective effect of CAR on subchondral bone. As showed in the figure, We found that the relative bone trabecular volume (BV/TV), trabecular separation(Tb.Sp), bone trabecular thickness (Tb.Th) and bone trabecular number (Tb.N) were reduced in the FAC group compared with the SHAM group(Fig. 1E). Copanied with the destroyed cartilage microstructure and the collapsed subchondral bone, these manifestations indicated that KOA had occurred in the FAC group mice. Whereas, the application of CAR treatment alleviates these changes. This suggests that CAR can slow down the deterioration of subchondral bone microarchitecture in iron-induced joint injury at both high and low concentrations.Indicating the potential use of CAR to treat this disease.

### CAR affects chondrocyte activity through dose-dependent

We measured the effect of CAR on chondrocyte activity by CCK8. The results showed that chondrocytes exhibited the greatest cell viability between the concentrations of 2.5–5 μm (Fig. 2B), so we took both concentrations of CAR, 2.5 μm and 5 μm; for the subsequent experiments. In addition, the cellular activity of chondrocytes after iron intervention was significantly reduced, and CAR was able to reverse this process (Fig. 2C). Toluidine blue staining showed that both 2.5 μM and 5 μM CAR reduced the impairment of FAC on the collagen synthesis capacity of chondrocytes, and there was no significant difference between the effect of 2.5 μM concentration and 5 μM concentration (Fig. 2D), which could also be verified on WB experiments: Matrix Metalloproteinase 3(MMP3) is a member of the matrix metalloproteinase family, which has a destructive effect on the extracellular matrix and other components of chondrocytes^22^; Collagen II (Col- II) is a macromolecular protein involved in the composition of the extracellular matrix of chondrocytes and is one of the main indicators for the evaluation of chondrocyte activity^23^. the CAR intervention significantly increased the level of Col-II and decreased the level of MMP3 in chondrocytes (Fig. 2E,F), and the PC group treated with DFO had almost the same effect. Thus, CAR had an inhibitory effect on chondrocyte damage caused by FAC.Chondrocyte differentiation is closely related to the ROS pathway and iron overload increases the production of ROS in cells, which accelerates the progression of OA^24^. Our flow and ROS fluorescence results show that intracellular ROS accumulate when chondrocytes are stimulated by FAC (Fig. 2G,H).

### CAR inhibits iron overload-induced chondrocyte apoptosis

**Figure 3:**
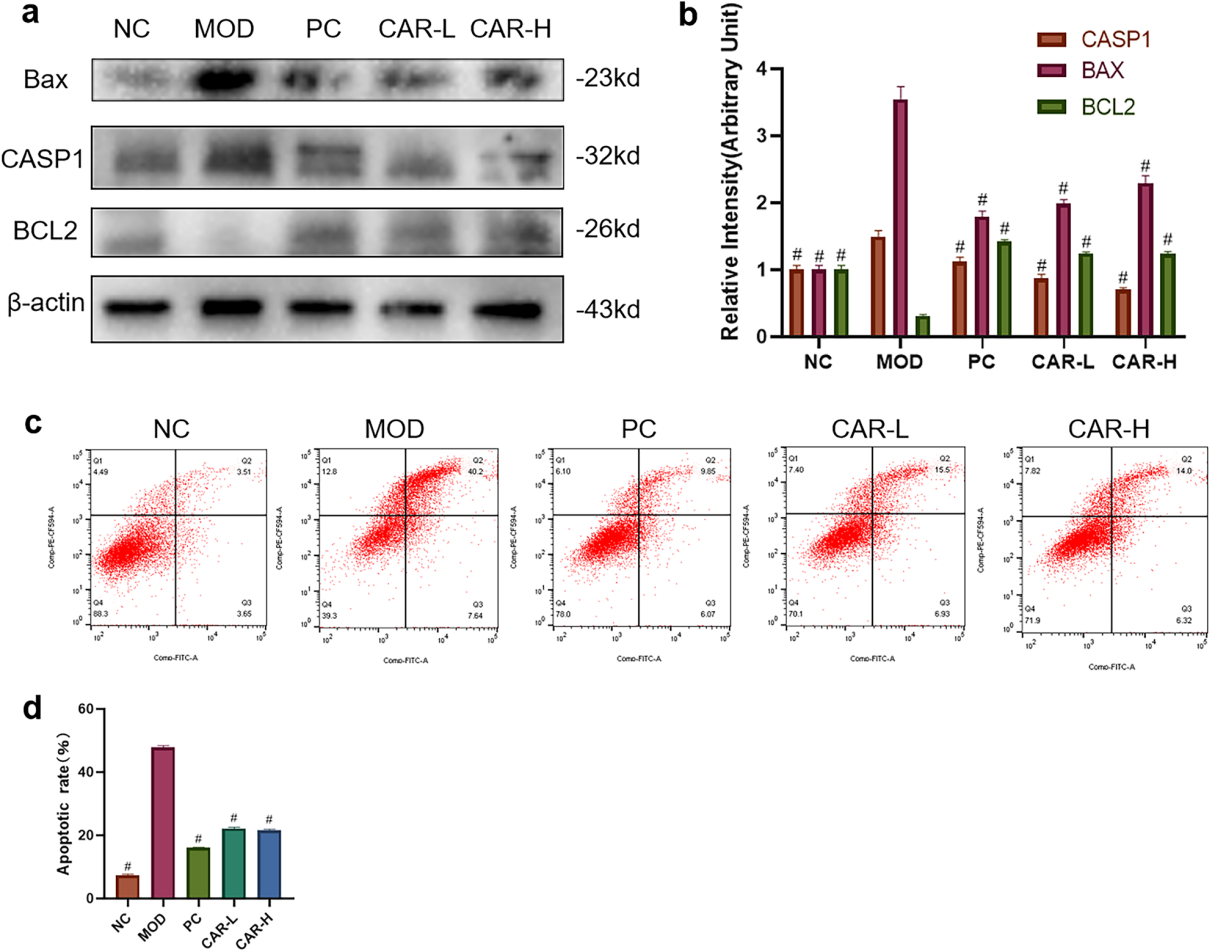
CAR helps to reduce iron overload-induced apoptosis in chondrocytes. (**a**,**b**) Chondrocytes were treated with 100 μm FAC and co-cultured with 0, 2.5, and 5 μm CAR, and BAX, CASP1, BCL2 protein levels were observed using protein blotting, and the statistical results are shown in the bar graph. (**c**,**d**) In addition, we examined changes in chondrocyte apoptosis under the same culture conditions using Annexin V-FITC/PI dual-labeling flow cytometry, and the results showed that CAR reduced FAC-induced chondrocyte apoptosis in a concentration-dependent manner. (data are presented as standard deviation ± mean; # *P* < 0.0001, *** *P* < 0.001, ** *P* < 0.01, * *P* < 0.05 vs. Mod).

To examine the protective effect of CAR on FAC-induced iron overload chondrocytes, we examined the levels of apoptotic factors by Western blot. The B-cell lymphoma-2(Bcl2) family is an important regulator of the endogenous pathway mediating apoptosis, and Bcl2 itself has an inhibitory effect on apoptosis^25^, while Bcl2 Associated X (BAX) proteins in the Bcl2 family bind to Bcl2 to form a dimer and inhibit its expression^26^. The Caspase family can directly disrupt cell structure and lead to apoptosis^27^. Intentionally, FAC intervention elevated BAX, Caspase1(CASP1), and decreased Bcl2 expression, while CAR intervention reversed this phenomenon (Fig. 3A,B); In addition, Annexin V-FITC/PI double-labeled flow cytometry also validated the results of Western blot experiments: CAR could alleviate the abnormal chondrocytes caused by FAC intervention apoptosis due to FAC intervention (Fig. 3C,D).

### CAR can reduce iron overload-induced IL-1β and IL-18 secretion via inhibiting NLRP3 expression

**Figure 4:**
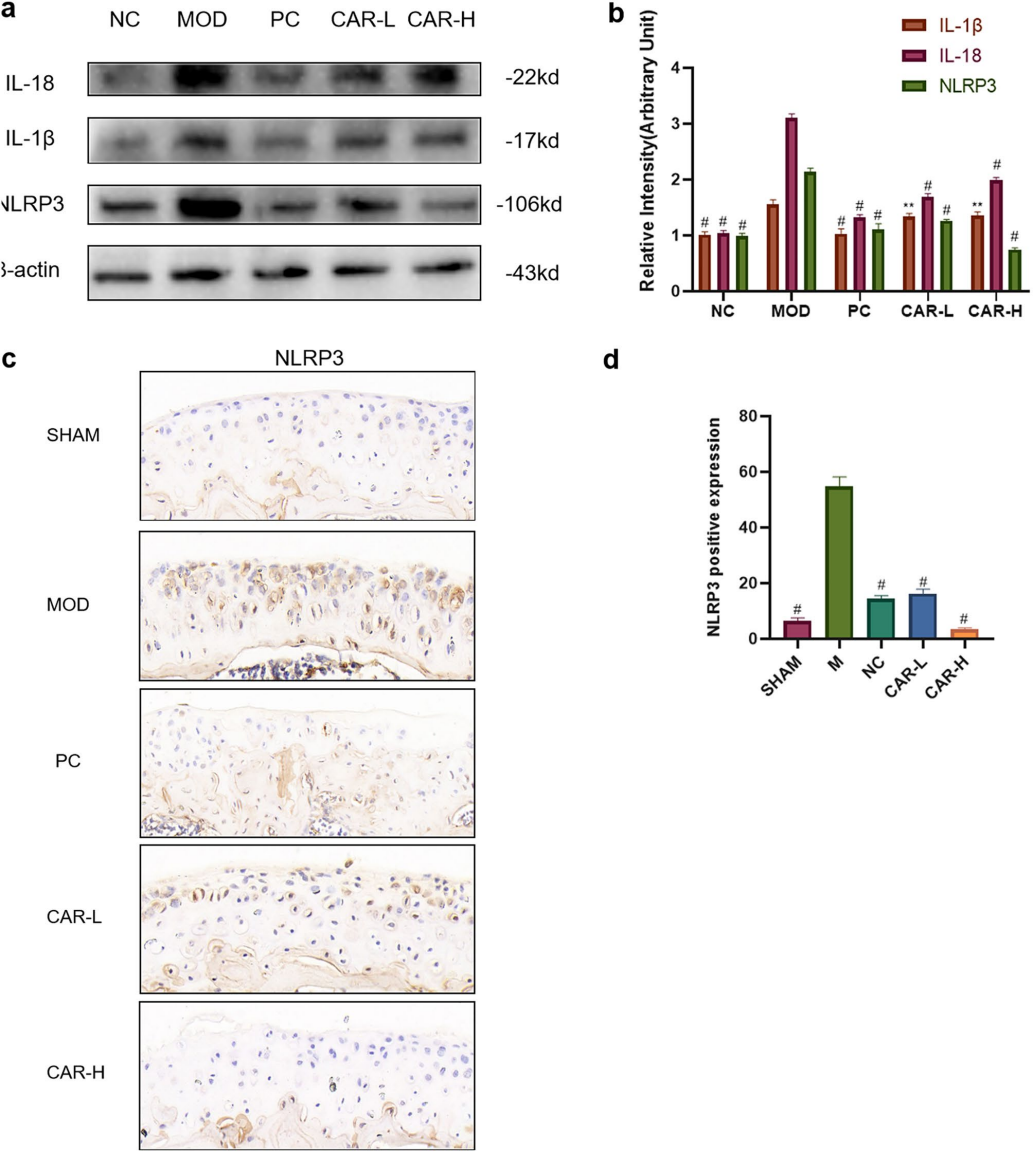
CAR helps to reduce iron overload-induced NLRP3 inflammasome vesicle production in chondrocytes. (**a**,**b**) Chondrocytes were treated with 100 μm FAC and co-cultured with 0, 2.5, and 5 μm CAR, and IL-18, IL-1β, and NLRP3 protein levels were observed using protein blots, and the statistical results are shown in the bar graph, which indicates that CAR reduced FAC-induced NLRP3 inflammasome production in a concentration-dependent manner. (**c**) Immunohistochemistry staining of NLRP3 (Scale bar = 200 μm). (**d**) Number of NLRP3 positive cells per area. (data are presented as standard deviation ± mean; # *P* < 0.0001, *** *P* < 0.001, ** *P* < 0.01, * *P* < 0.05 vs Mod).

To test whether CAR can affect the expression of inflammatory specific factors in FAC-induced chondrocytes, we examined the expression of Interleukin -1β(IL-1β) versus IL-18 in chondrocytes using Western blot assays. The data showed that FAC induced a significant increase in IL-1β and IL-18 secretion by chondrocytes, and CAR pretreatment inhibited the release of both pro-inflammatory cytokines in a dose-dependent manner (Fig. 4A,B). Therefore, we hypothesized that CAR inhibited NOD-like receptor protein 3(NLRP3) inflammasome specific cytokine secretion, and this effect was achieved by suppressing IL-1β and IL-18 gene expression. The results of western blot experiments proved our conjecture that the increased IL1-β and IL-18 secretion promotes the activation of NLRP3 inflammasome while CAR reverses this process (Fig. 4A,B). We also verified the above results in vivo. Immunohistochemistry revealed that NLRP3 protein expression in the model group was increased compared to the sham group.

### CAR reduces NLRP3 inflammasome expression and chondrocyte apoptosis by inhibiting p38MAPK pathway through activation of SIRT1

**Figure 5:**
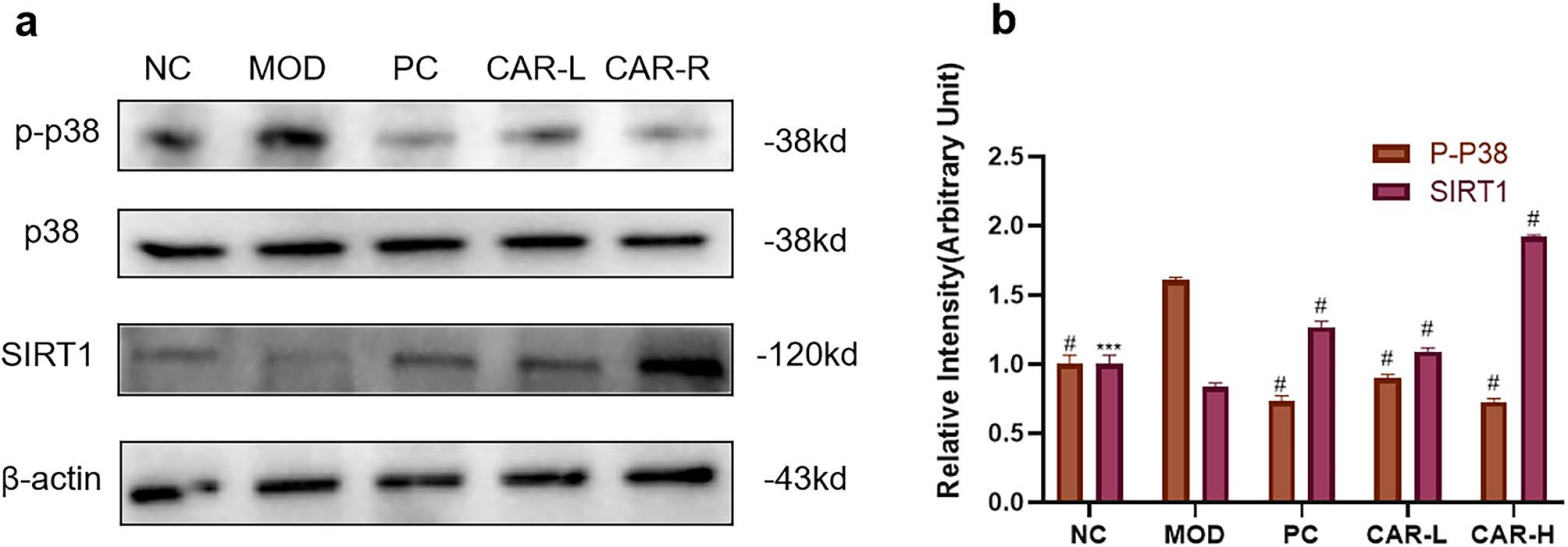
CAR inhibits MAPK signaling expression through activation of SIRT1. (**a**,**b**) Chondrocytes were treated with 100 μm FAC and co-cultured with 0, 2.5, and 5 μm CAR, and p38, p-p38, and SIRT1 protein levels were observed using protein blots, and statistical results are shown in bar graphs (data are presented as standard deviation ± mean; # *P* < 0.0001, *** *P* < 0.001, ** *P* < 0.01, * *P* < 0.05 vs. Mod).

To further reveal the mechanism of CAR inhibition of NLRP3 inflammasome expression and chondrocyte apoptosis, we examined p38MAPK signaling as well as SIRT1 expression using Western blot assay. FAC significantly increased the expression of p-p38. Compared with the FAC intervention group, CAR effectively reduced the expression level of p-p38 protein, while the expression of SIRT1 was significantly higher than that of the FAC intervention group (Fig. 5A,B). Thus, we found that CAR inhibited the expression of p38MAPK signaling through activation of SIRT1.

### SIRT1-inhibitor EX-527 eliminate the inhibitory effect of CAR on FAC-induced NLRP3 inflammasome expression and chondrocyte apoptosis

**Figure 6:**
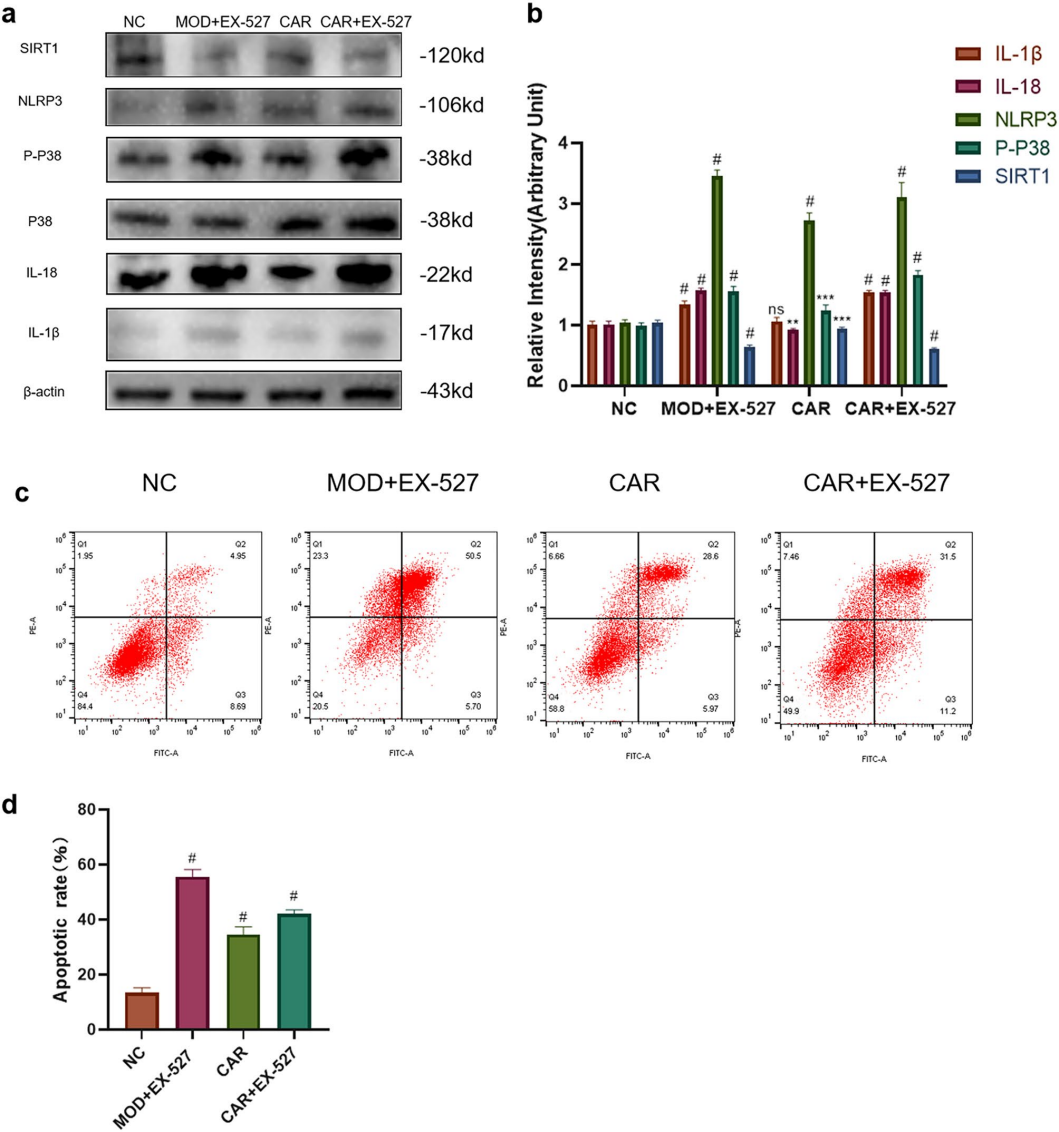
SIRT1 inhibitor EX527 reverses the inhibitory effect of CAR on FAC-induced chondrocyte apoptosis and NLRP3 inflammasome secretion. (**a**,**b**) Chondrocytes were treated with 100 μm FAC and co-cultured with 10 μm EX527, 5 μm CAR, 5 μm CAR + 10 μm EX527. And p38, p-p38, SIRT1, IL-18, IL-1β, NLRP3 protein levels were observed using protein blotting and the statistical results are shown in the bar graphs. (**c**,**d**) In addition, we examined changes in chondrocyte apoptosis under the same culture conditions using Annexin V-FITC/PI dual-labeling flow cytometry (data are shown as standard deviation ± mean; # *P* < 0.0001, *** *P* < 0.001, ** *P* < 0.01, * *P* < 0.05 vs. Mod).

To investigate whether CAR inhibits NLRP3 inflammasome expression and chondrocyte apoptosis by regulating the SIRT1/p38MAPK pathway, we divided the cells into NC, MOD + EX527, CAR, and CAR + EX527 groups, then pretreated chondrocytes for 2 h. By Western blot assay we found that EX-527 did not reverse FAC-induced chondrocyte apoptosis and NLRP3 inflammasome expression, but the inhibitory effects of CAR on IL-1β, IL-18, NLRP3, p-p38 were counteracted by EX-527 (Fig. 6A,B). In addition, Annexin V-FITC/PI dual-labeling flow cytometry also suggested that EX-527 counteracted the inhibitory effect of CAR on chondrocyte apoptosis caused by FAC intervention (Fig. 6C,D).

## Discussion

Iron is an essential trace element in humans.In addition to being an essential component of heme, iron also participates in various cellular functions, such as oxygen transport, energy metabolism, and detoxification through catalytic oxidation–reduction reactions^28^. In the body of divalent iron and trivalent iron are present in large quantities, through the combination with transferrin to form the iron-transferrin complex (Fe-TF), Fe-TF and the transferrin receptor 1 (TFR1) on the membrane of the target cell to form the TFR1-Fe-TF complex is endocytosed into the cell, into the cell only to be reduced to the divalent iron into the cytoplasm, the cellular organelles, such as into mitochondria Participate in metabolism synthesizing hemoglobin, etc.^29^. Studies have demonstrated that high iron levels cause ROS accumulation and inflammatory responses in chondrocytes^24,30^. This study reveals the important role of CAR in the treatment of cartilage degeneration and KOA progression. CAR inhibited the MAPK pathway and ROS by activating SIRT1, thus suppressing NLRP3 inflammatory secretion and chondrocyte apoptosis.

We first established an iron overloaded KOA mouse model by the intraperitoneal injection of iron dextran combined with DMM surgery^31,32^. Compared to the sham group, Prussian blue staining of synovial tissue in the mod group demonstrated substantial iron deposition. The use of positive drugs and CAR significantly reduced intra-articular iron deposition. And our Safranin O and HE staining results revealed that CAR could also rescue cartilage destruction caused by iron overload. Abnormal formation of the subchondral bone is caused by uncoupled bone remodeling and is considered a central feature of KOA pathogenesis^3^. We used micro-CT to quantify structural changes in the tibial subchondral bone in an iron-overload-induced KOA mouse model. Our results revealed that the model group had significantly reduced number of bone trabeculae in the subchondral bone and significantly increased degree of bone trabecular separation compared to those in the control group, and CAR reduced the changes in bone morphological structure and bone microstructure coefficient of mice. Therefore, we believe that CAR can alleviate in vivo subchondral bone loss and degeneration caused by iron overload in early KOA, thereby limiting OA progression.

Based on the in vivo results, we further investigated the role of CAR in vitro. The degree of cartilage and stromal destruction in OA patients is positively correlated with the degree of chondrocyte apoptosis^33^. In the present study, we used FAC to construct a chondrocyte iron overload model to simulate an in vitro pathological environment. Through toluidine blue staining and flow cytometry, we first found that 5 and 10 μM CAR could save chondrocyte apoptosis and reduce ROS production in iron overload environment, and further wb experiments verified that CAR could promote Col-II expression and inhibit MMP3 expression in chondrocytes. However, the detailed mechanisms remain unclear. Studies have indicated that iron overload inhibits SIRT1 expression and plays a key role in KOA progression^34,35^. We also found that iron overload can inhibit the expression of SIRT1 and BCL2, and promote the expression of BAX and CASP1. Our WB results showed that CAR could activate the expression of SIRT1. In addition, it has been reported that decreased SIRT1 expression leads to increased ROS levels in chondrocytes^36^. Combined with our experimental results, CAR regulates ROS levels in chondrocytes, which may be related to the activation of SIRT1 by CAR. Therefore, CAR may protect chondrocytes from iron overloading induced apoptosis through SIRT1 signaling pathway.

Previous studies have reported that the NLRP3 inflammasome mediates the maturation of pro-inflammatory cytokines IL-1β and IL-18, which may be the cause of KOA^37^. Here, we observed increased NLRP3 expression in chondrocytes under an iron-overload environment, along with the increase of IL-1β and IL-18 expressions. After CAR intervention, NLRP3 expression was inhibited.However, the exact mechanism by which NLRP3 expression is suppressed remains unclear. It has become a consensus SIRT1 can regulates the expression of the p38MAPK pathway affecting NLRP3^38–40^. In the previous study, we observed that CAR increases SIRT1 expression and inhibits p38 phosphorylation. Thus, we suggest that CAR can also inhibit NLRP3 to protect chondrocytes through the SIRT1/p38MAPK signaling pathway. For further verification, we pharmacologically inhibited SIRT1 activity using EX-527, a SIRT1 inhibitor. The WB results suggested that EX-527 reversed the inhibitory effect of CAR on NLRP3 and apoptosis, indicating that CAR may protects chondrocytes by inhibiting NLRP3 inflammasome production through the SIRT1/p38MAPK pathway.

## Conclusion

**Figure 7:**
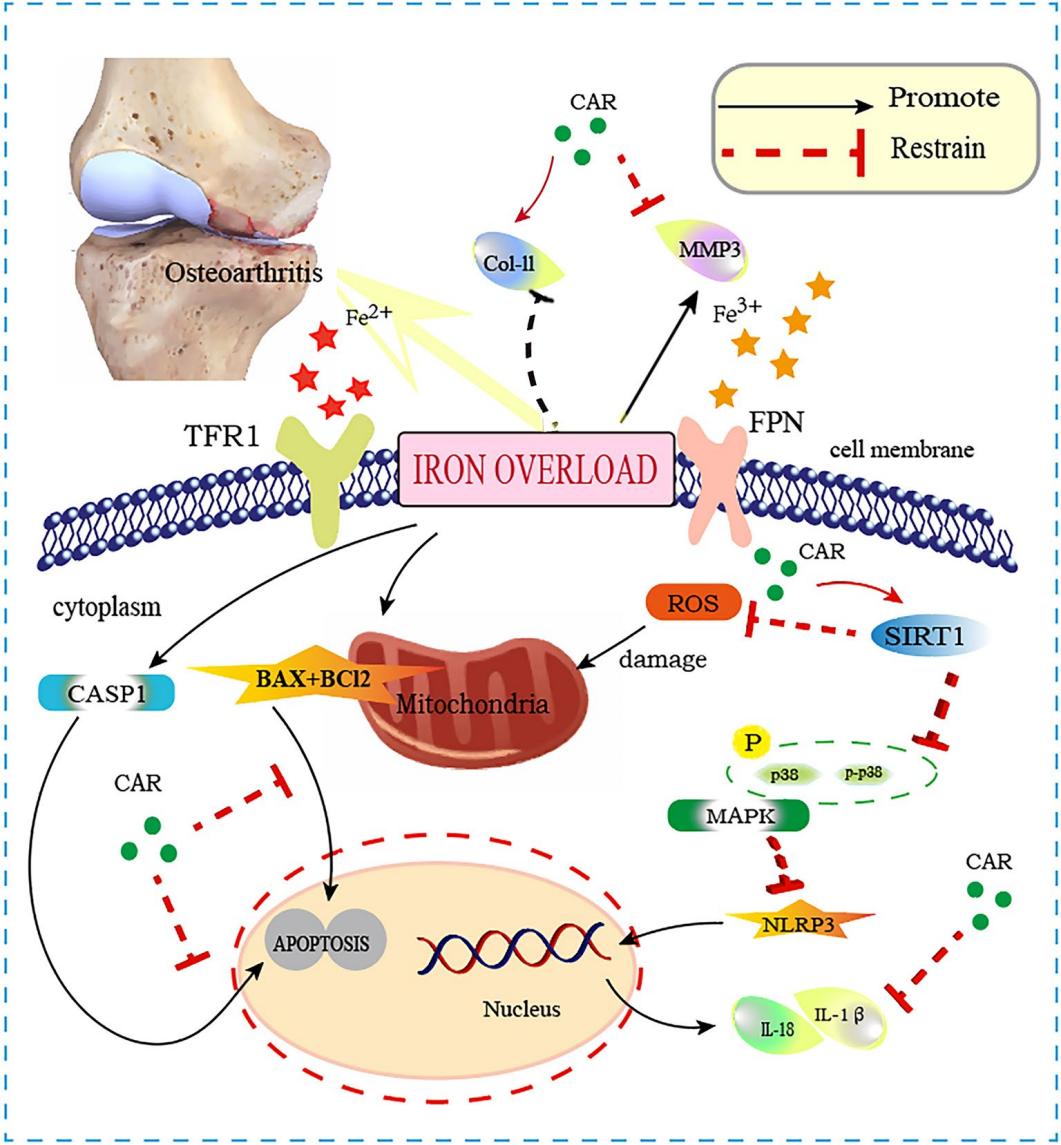
A graphical abstract for illustrating the role of CAR on SIRT1/MAPK/NLRP3, apoptosis. Iron overload can inhibit the expression of SIRT1 in chondrocytes, promoting the increase of ROS levels and the expression of NLRP3 inflammasome corpuscle, and ultimately inducing chondrocyte apoptosis.

Overall, there is a strong association between iron overload and the incidence and progression of KOA. According to our results, iron overload can inhibit the expression of SIRT1 in chondrocytes, thereby promoting the increase of ROS levels and the expression of NLRP3 inflammasome corpuscle, and ultimately inducing chondrocyte apoptosis. CAR is a compound extracted from the ginger family that exhibits significant antioxidant and anti-inflammatory properties in several diseases. Our results suggest that CAR can activate SIRT1 to directly inhibit ROS production and apoptotic gene expression, and reduce the expression of inflammatory cytokines through the p38MAPK/NLRP3 signaling pathway. Therefore, the results of this study suggest that CAR can regulate the SIRT1/p38MAPK signaling pathway to inhibit ROS production and NLRP3 inflammasome activation for the treatment of iron overload osteoarthritis, which will open up a new field for the treatment of KOA(Fig. 7).

### Supplementary Information

Supplementary Figures.

## Data Availability

The datasets generated and/or analyzed during the current study are available from the corresponding author on reasonable request.
