# Supplementary material for: Cardamonin protects against iron overload induced arthritis by attenuating ROS production and NLRP3 inflammasome activation via the SIRT1/p38MAPK signaling pathway
Source: Sci Rep. 2023 Aug 23;13:13744. doi: 10.1038/s41598-023-40930-y (PMC10447427; doi:10.1038/s41598-023-40930-y)

figure2 mmp3

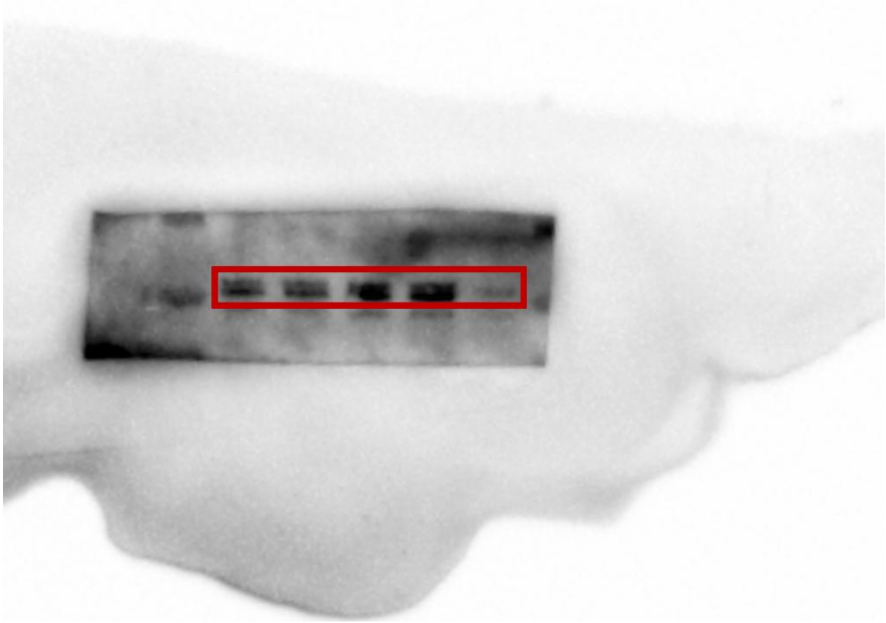

figure2 β-actin

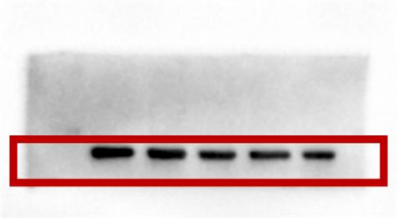

figure2 col2

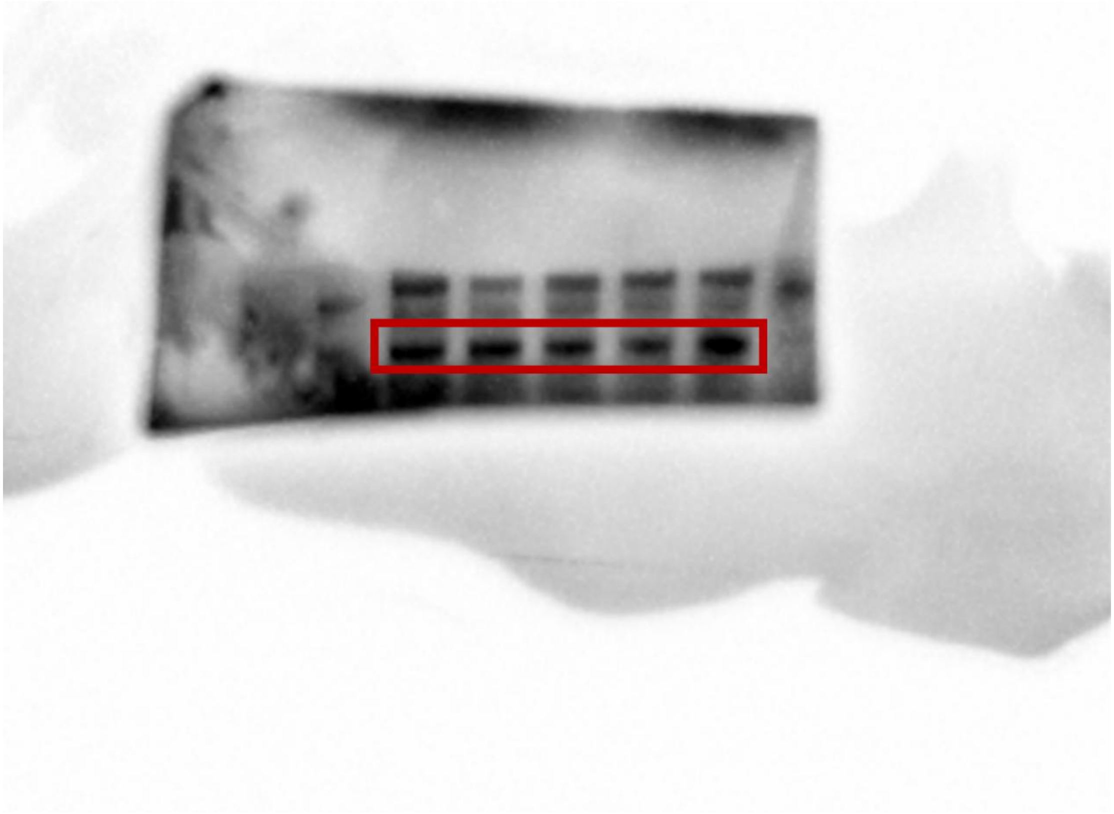

figure3 bax

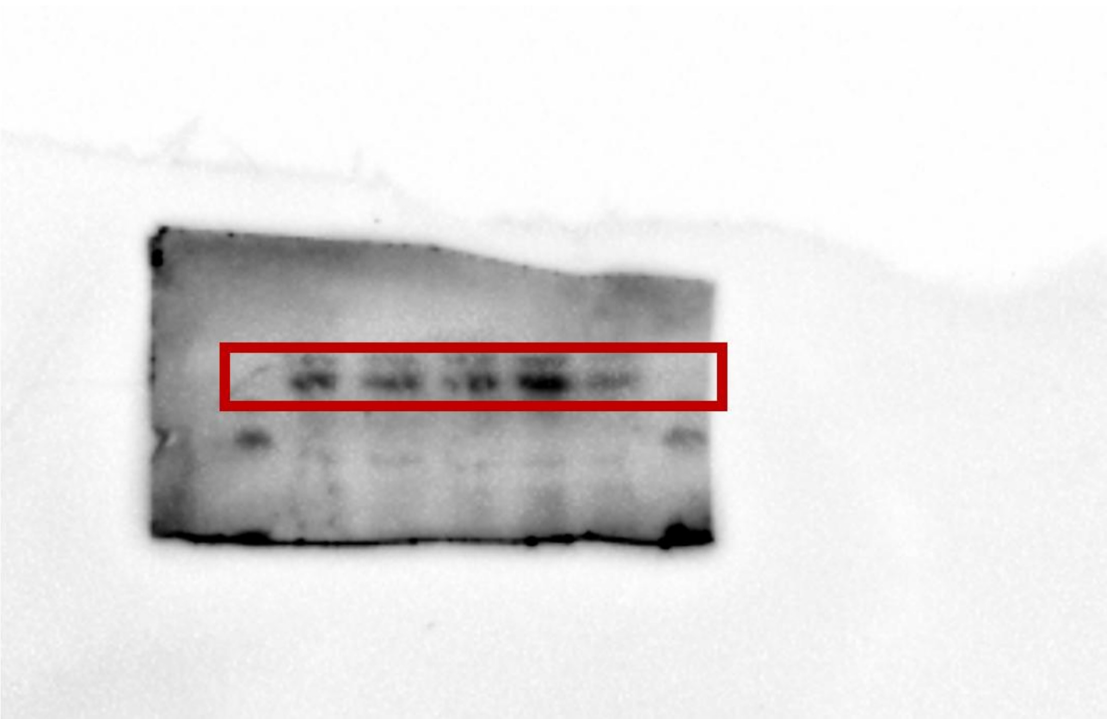

figure3 bcl2

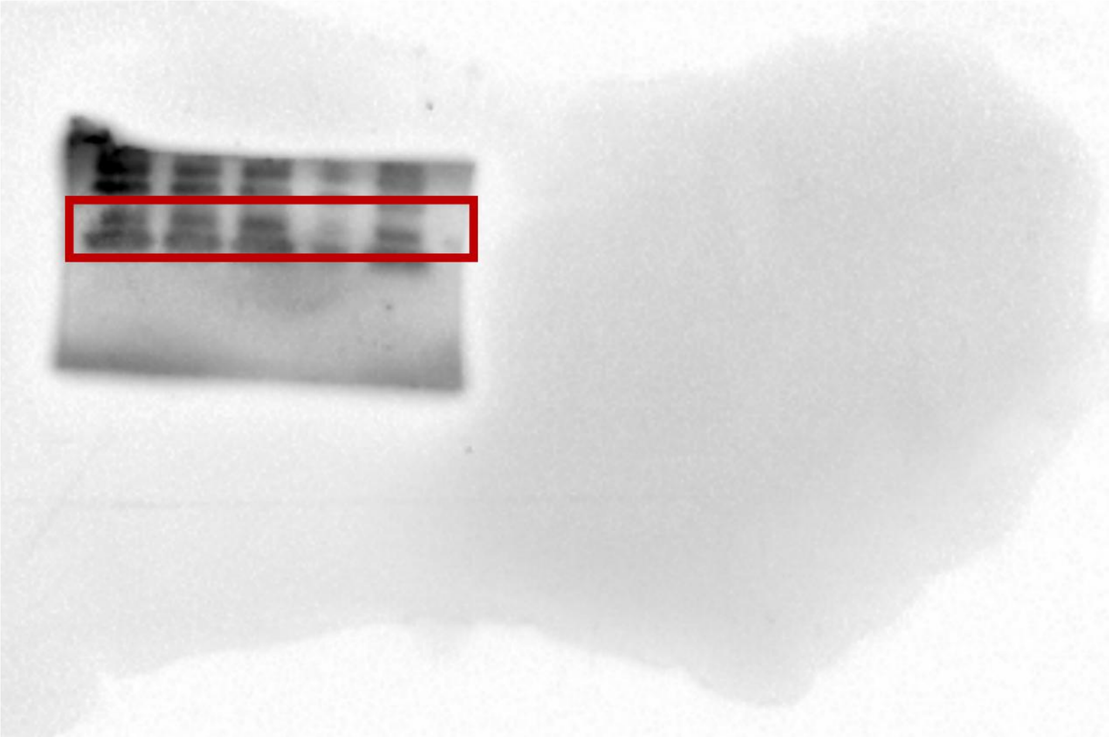

figure3 casp1

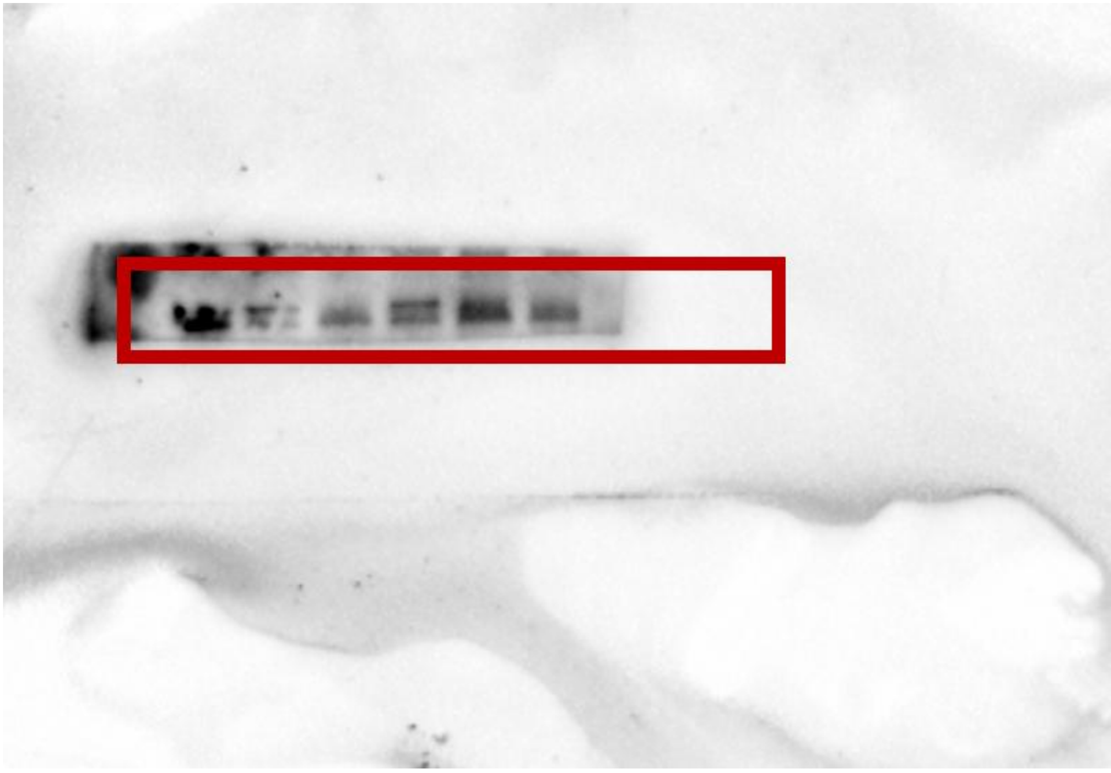

figure3 β-actin

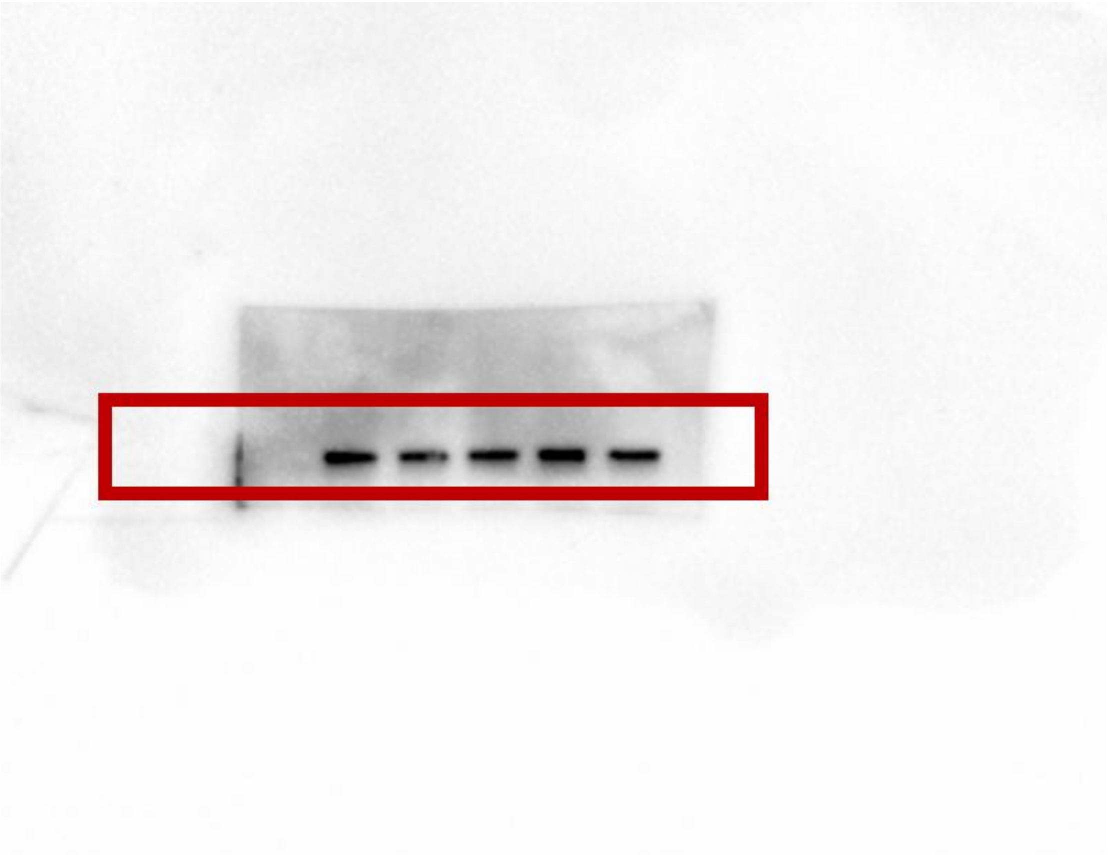

figure4 figure5 β-actin

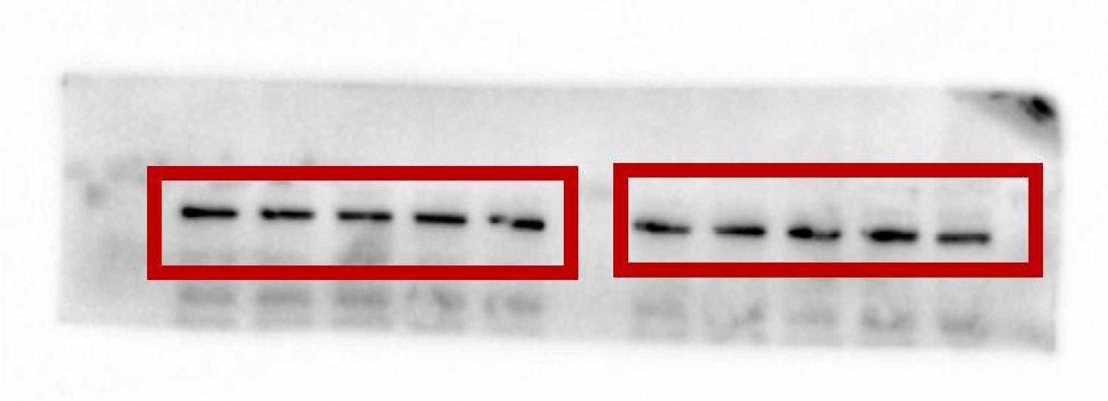

figure4 IL1β

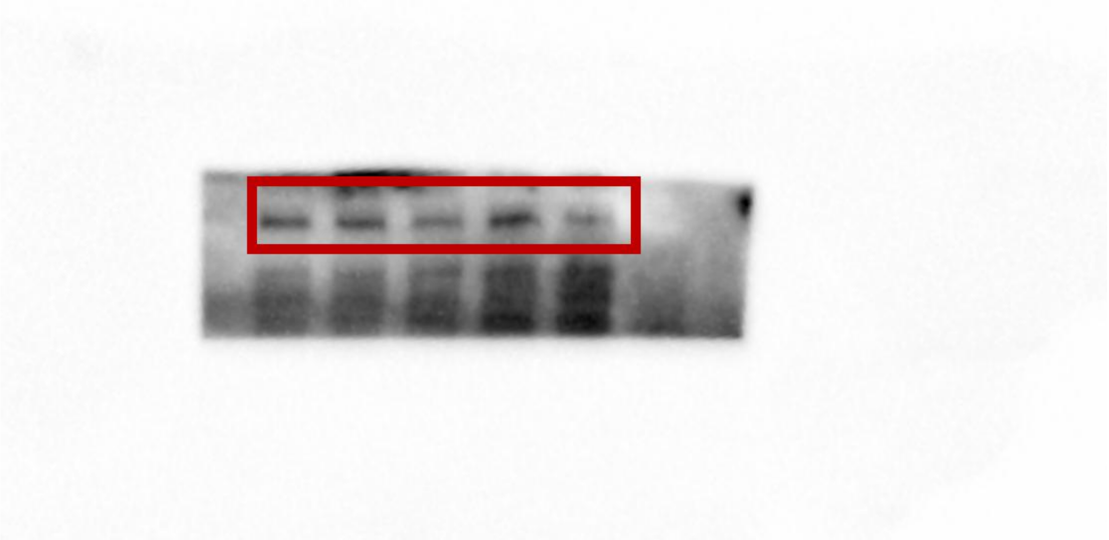

figure4 IL18

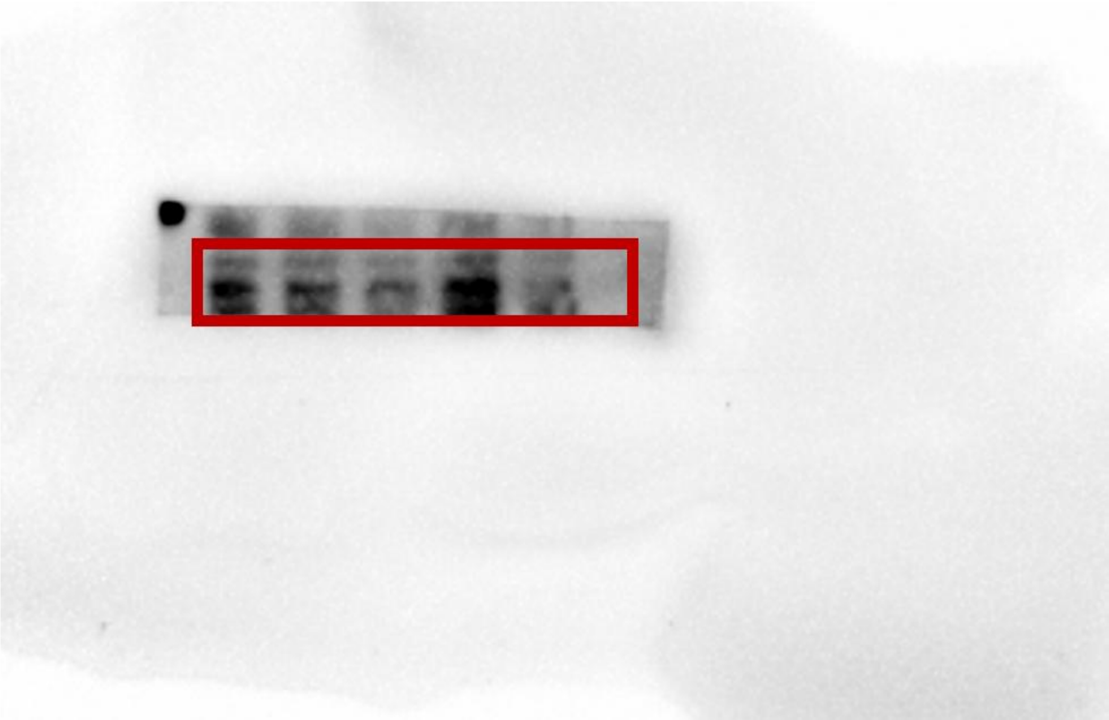

figure4 NLRP3

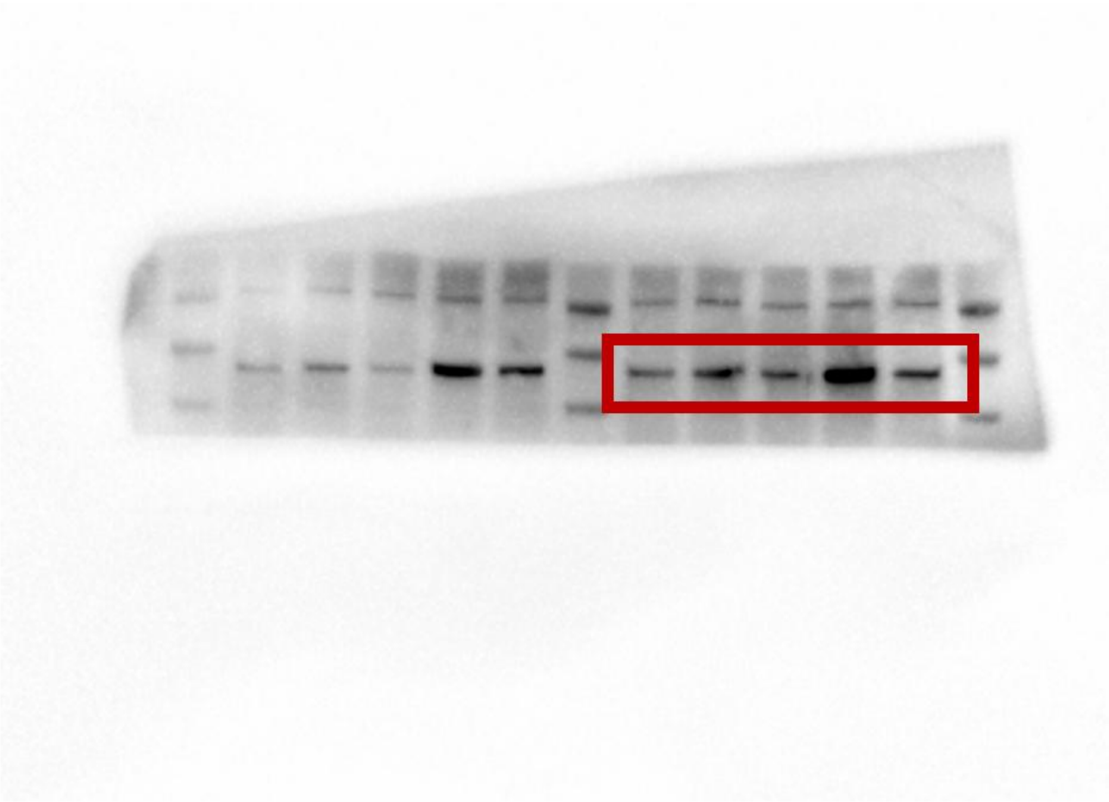

figure5 P38

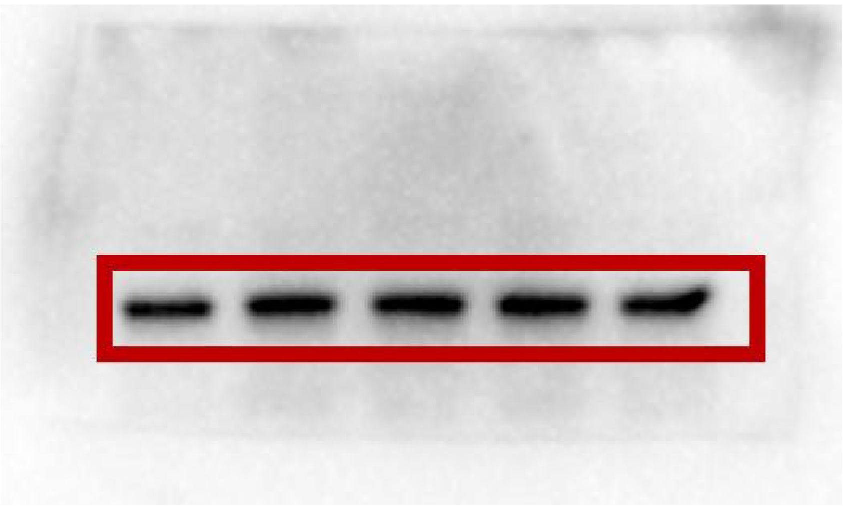

figure5 p-P38

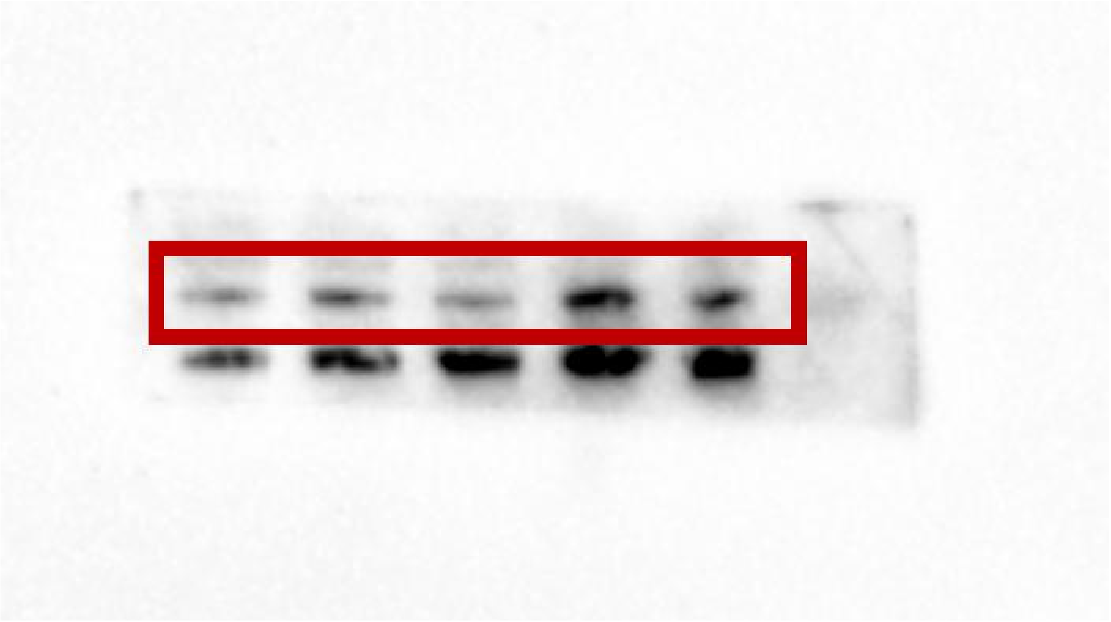

figure5 SIRT1

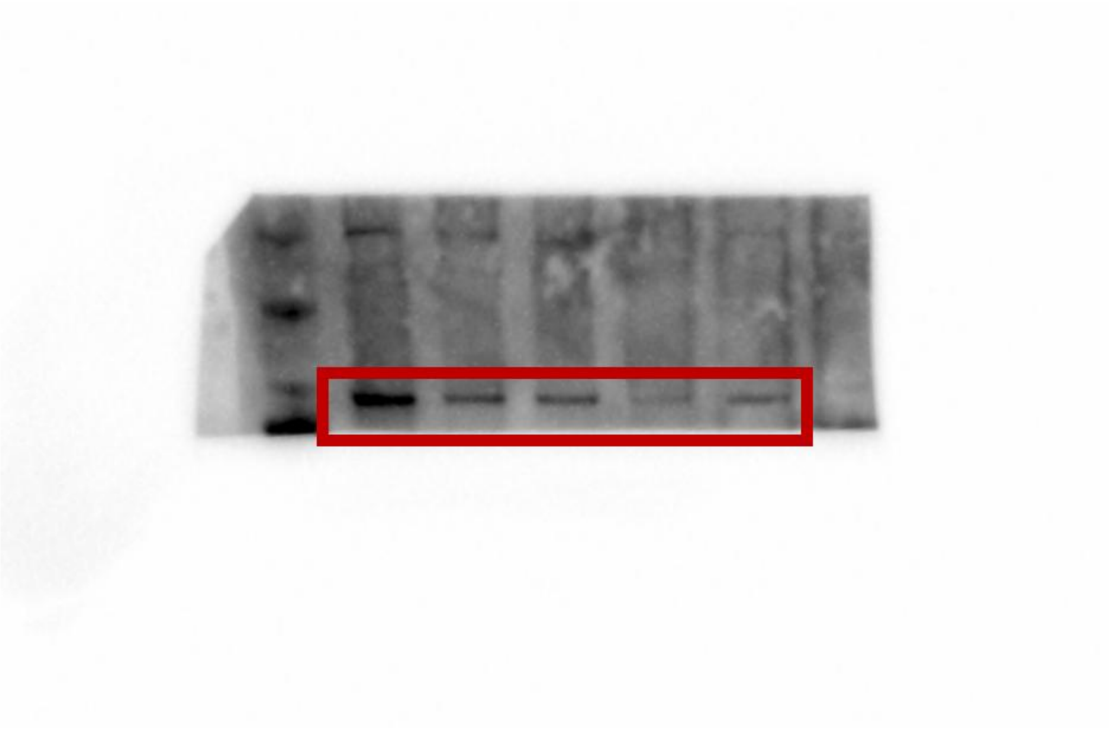

figure6 IL1β

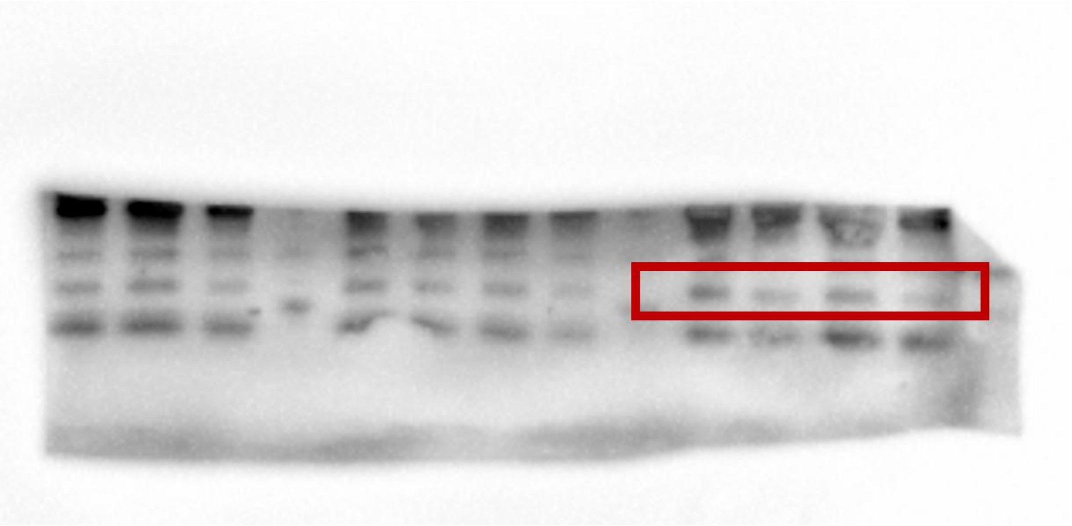

figure6 IL18

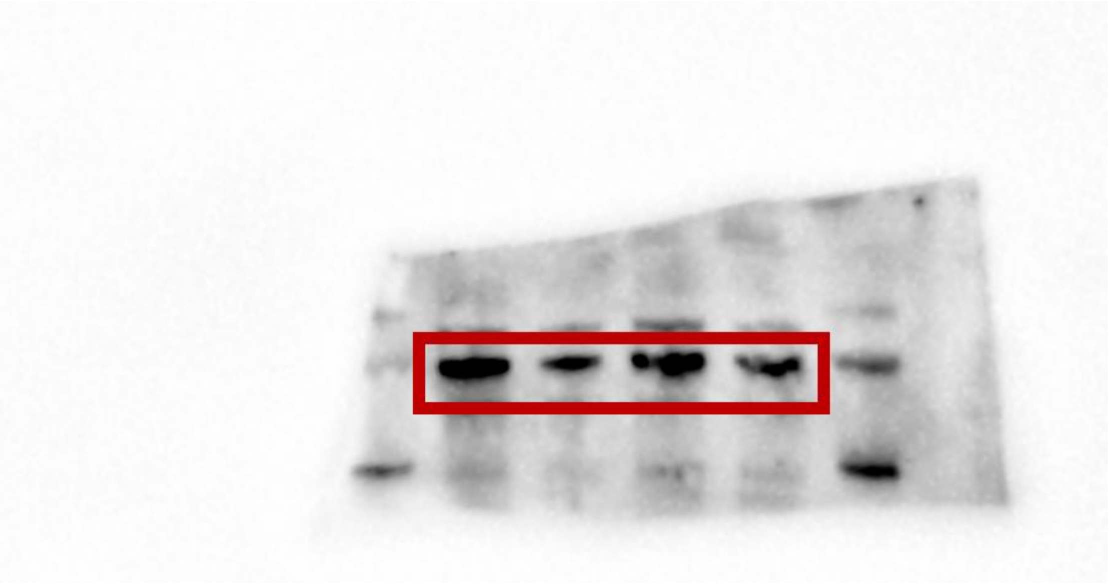

figure6 NLRP3

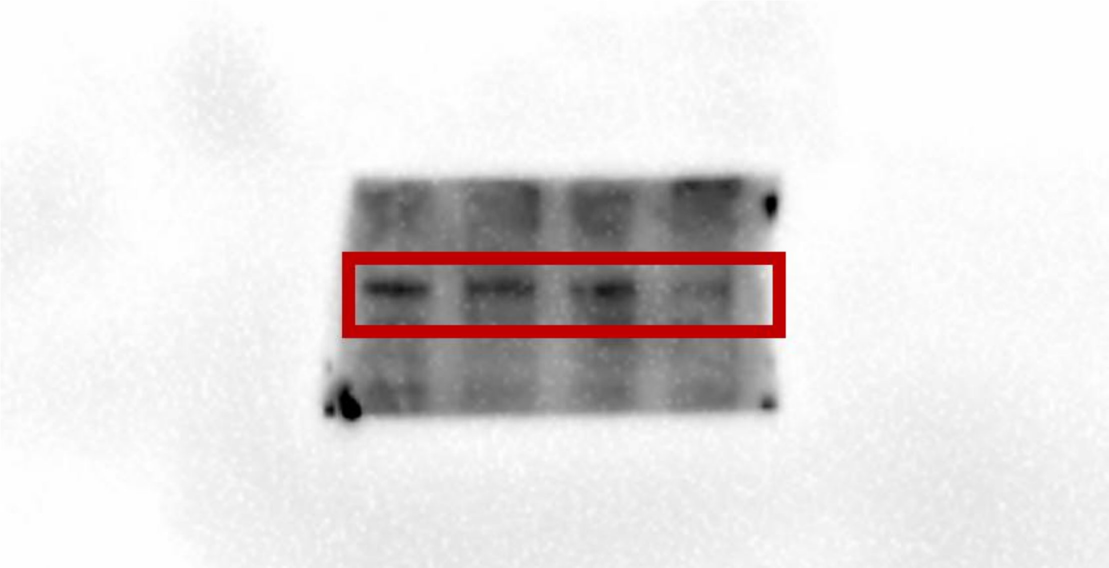

figure6 P38

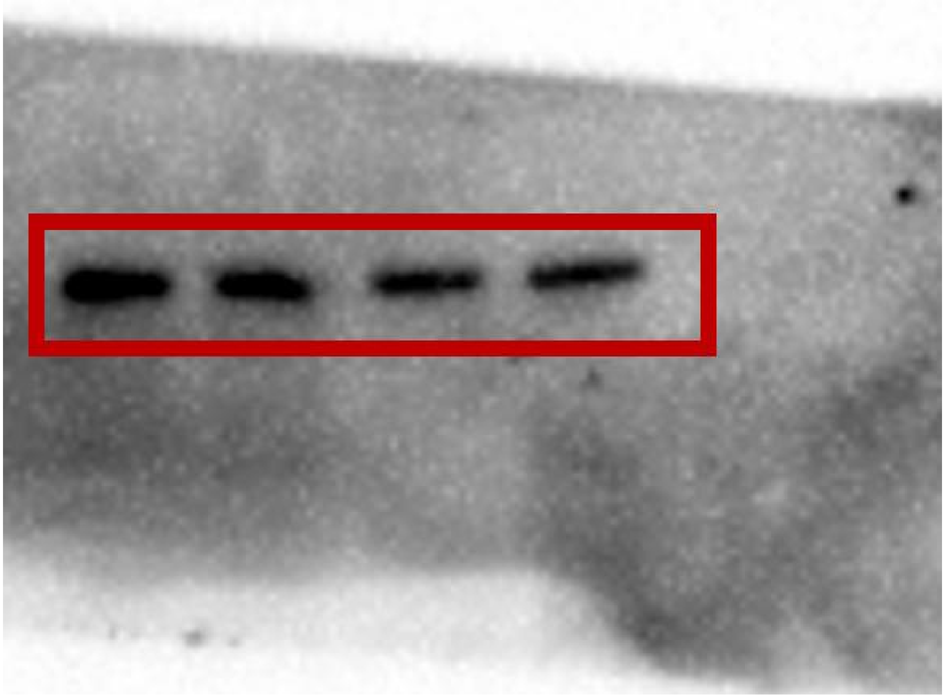

figure6 p-P38

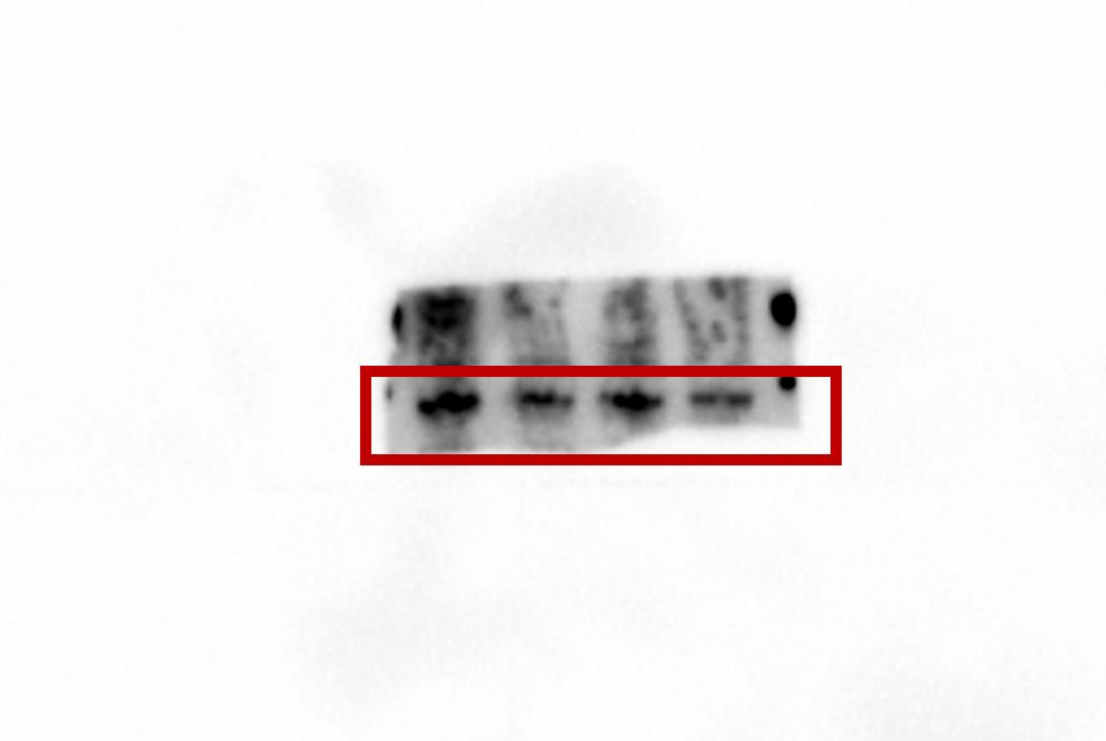

figure6 SIRT1

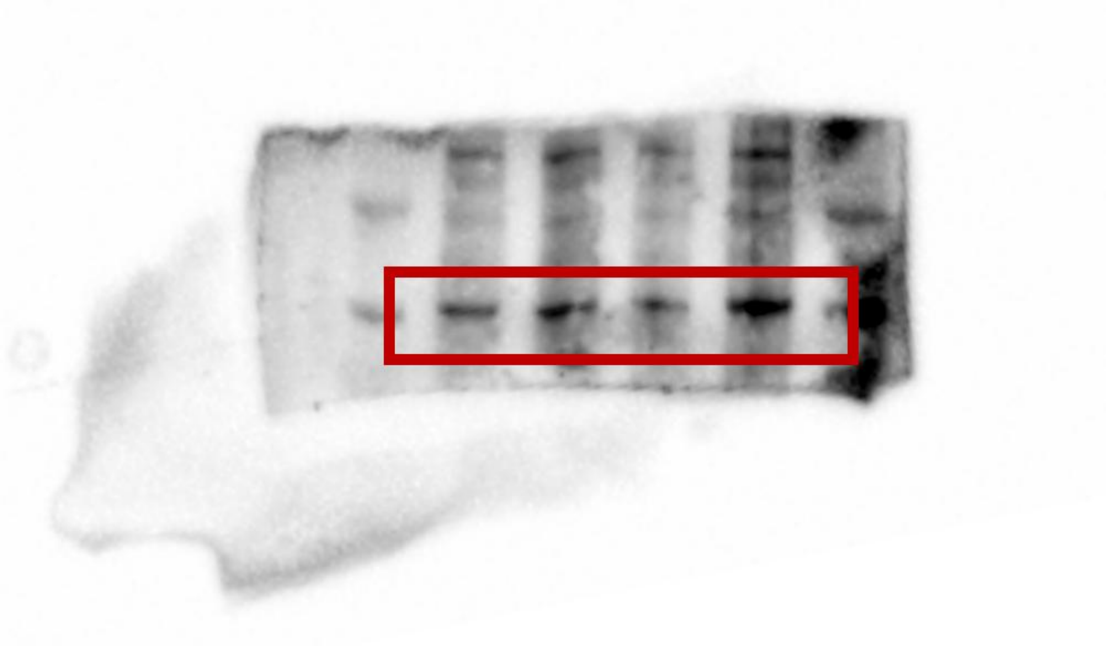

figure6 β-actin

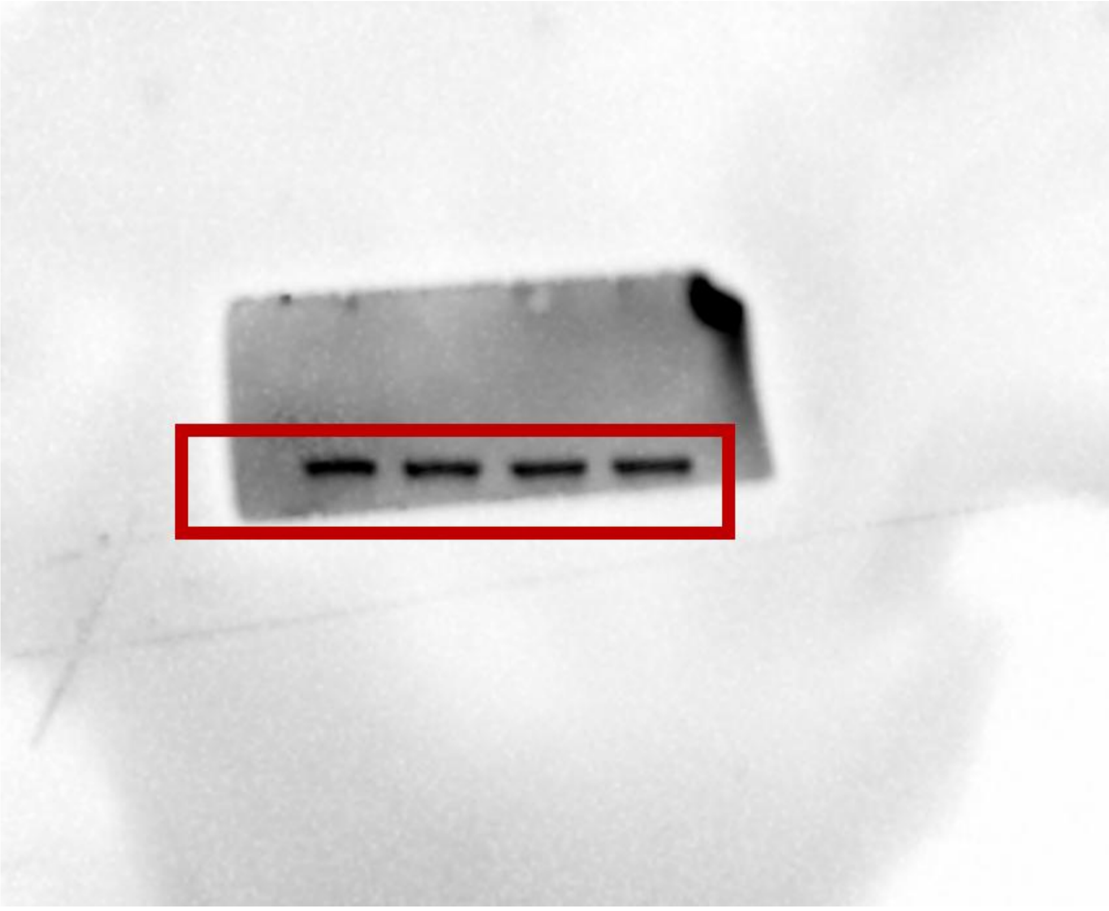

Supplement: Supplementary file 1 — Supplementary Figures. [file 41598_2023_40930_MOESM1_ESM.pdf]
